# Leednet: a lightweight network for event detection in EEG signals

**DOI:** 10.1186/s13634-026-01330-2

**Published:** 2026-05-26

**Authors:** Mohammad Ali Alqarni, Hira Masood, Hassan Aqeel Khan, Awais Mehmood Kamboh, Saima Shafait, Tooba Jaffarey, Faisal Shafait

**Affiliations:** 1 College of Computer Science and Engineering, University of Jeddah, Jeddah, Saudi Arabia; 2 School of Electrical Engineering and Computer Science, National University of Sciences and Technology, Islamabad, Pakistan; 3 Deep Learning Laboratory, National Center of Artificial Intelligence, Islamabad, Pakistan; 4 Department of Applied Artificial Intelligence and Robotics, Aston University Birmingham, Birmingham, B4 7ET United Kingdom; 5 Pak-Emirates Military Hospital, Rawalpindi, Pakistan; 6 AI Lounge Private Limited, Islamabad, Pakistan; 7 Guangdong CAS Cogniser Information Technology Co., Ltd., Guangzhou, China

**Keywords:** Automated EEG analysis, Computer-aided diagnosis, Computational neurology, Convolutional neural networks, Deep learning

## Abstract

Detection of diagnostically relevant events within an EEG recording requires making classification decisions about small event windows of a few seconds duration each. For long-duration EEG recordings, this can translate into thousands of decisions leading to a high computational load, which must be reduced for field deployments of EEG triage and decision support systems, especially in resource-constrained healthcare systems. In this work, we explore three avenues for enhancing computational efficiency: (1) purpose-built architectures; (2) length of the decision window/segment; and (3) lightweight preprocessing and feature extraction. For the architecture, we present LEEDNet (Lightweight EEG Event Detection Network), a lightweight convolutional architecture that is purpose-built for prompt classification of short EEG events into three categories (Normal, Slow Waves, or Spike and Sharp Waves). LEEDNet is evaluated on the expert-annotated NMT-Events dataset with subject-wise splits. Compared to four state-of-the-art baselines, LEEDNet delivers the best performance, on three out of four, performance evaluation metrics while being much more deployment friendly (requiring only 0.25 M parameters and 37 MFLOPs per segment, enabling inference in < 5 ms on a standard CPU). For the segment length, ablation results show a length of 2 s to be the best performing, with longer segments degrading due to label dilution and background dominance. We also examine the impact of different feature engineering configurations and whether the increased computational overhead associated with extraction of complex features leads to substantial performance gains. In particular, we examine three input modalities—raw signals, FFT-based spectra, and wavelets—both independently (single-branch) and in a modular, multi-branch fusion model. Our results indicate that the single-branch LEEDNet, based on Raw EEG data, achieves the best overall performance, with 81.2% accuracy and 77.5% macro-F1. No consistent gains were observed after the introduction of modalities based on Fourier and Wavelet features indicating that, for our architecture, a data-driven approach (based on raw EEG waveforms) is better suited compared to a handcrafted feature engineering approach. This is welcomed from a computation overhead perspective since complex feature engineering can be eliminated in favor of raw samples as input features without sacrificing performance.

## Introduction

Neurological disorders such as epilepsy, encephalopathy, and neurodegenerative diseases affect over one billion people worldwide [1]. The burden of these conditions is particularly high in low- and middle-income countries (LMICs), where specialized neurological care is scarce [2]. Many regions face a critical shortage of neurologists, limited neuroimaging capacity, and inadequate access to continuous monitoring. Given these challenges, electroencephalography (EEG) becomes an important tool for non-invasive assessment of cerebral function due to its low-cost [3]. EEG is especially valuable in low-income and rural areas where MRI or CT may be inaccessible. Portable EEG systems enable remote assessments in community clinics, mobile units, and telemedicine frameworks. According to estimates by the WHO, many LMICs have fewer than one neurologist per 100,000 people [2], indicating that scalable diagnostic solutions are desperately needed. Despite its affordability and diagnostic potential, EEG interpretation is labor-intensive and requires expert-level training. A standard recording may span 20–30 min (or longer) with 21 or more channels, sampled at high frequencies. Detecting transient abnormalities—such as epileptiform discharges, focal or generalized slowing, sharp waves, or spike-and-wave complexes—requires detailed segment-wise inspection. This process is time-consuming, subject to inter-rater variability [4, 5], and increasingly impractical in high-throughput settings such as ICUs and tele-neurology programs. Field deployments in LMICs often require the use of low-cost, battery-operated hardware since power outages are common and long lasting. Consequently, the computational overhead of any automated screening and decision support systems must be kept low.

Traditional EEG classification systems rely on handcrafted features such as spectral entropy, wavelet energy, or Hjorth parameters [6], often processed using classical machine learning methods like support vector machines (SVMs), decision trees, or random forests [7, 8]. However, such systems are typically task-specific, sensitive to noise, and limited in their ability to generalize across patients or clinical protocols. Recent deep learning (DL) methods—including EEGNet [9], ChronoNet [10] and DeepConvNet [11]—have shown that neural networks can learn robust temporal and spatial patterns directly from raw EEG data or with minimal preprocessing. While effective for seizure detection, BCI control, or sleep staging, many of these models operate at the recording level, providing only binary predictions without indicating when or where an abnormality occurs. This limits their usefulness in real-world settings, where clinicians review EEG in short segments, assessing each window for specific pathological events. More recently, in CTNet [12] and TCANet [13], convolutional transformer networks were used for classification of short (∼6-sec) EEG segments was explored but focus was on motor imagery classification tasks instead of clinically relevant events. Furthermore, an extensive evaluation of computational performance by the authors in [13] demonstrated that computational overhead was higher compared to lightweight, CNN-based, baseline architectures.

Segment-level classification and event localization have, therefore, emerged as more clinically relevant goals. This was addressed in [14] by introducing the NMT-Events dataset, which contains detailed annotations of events of interest, such as slow waves (SW) and spike and sharp waves (SSW) at a 2-second resolution. However, the computational overhead associated with this pipeline is quite high. In [15], a hybrid deep learning approach based on a combination of a residual and bidirectional LSTM was proposed for epileptic seizure detection in small (∼3-sec) EEG segments. Although, this architecture is efficient and outperformed a number of SOTA architectures, computational efficiency was not formally evaluated by the authors. There are three avenues within the segment-level classification pipeline where computational efficiency can be introduced: (1) deep learning architecture design; (2) preprocessing and feature extraction; (3) segment length adjustment of the (classification) decision window. The computational overhead associated with predictions from a typical deep learning architecture can be significant due to the large size of the network. Preprocessing and feature extraction add to this overhead, especially when the number of segments to be classified is large. The use of small segment lengths increases the number of classification decisions that need to be made; thus, increasing segment length is also appealing; however, longer segments imply lower temporal resolution, making it difficult to localize or differentiate between short-duration events. In this work, we examine all three of the preceding avenues to enhance the computational efficiency of the pipeline in [14] without compromising performance. The main contributions of this work are:

- We introduce LEEDNet (Lightweight EEG Event Detection Network), a lightweight deep learning model for classifying EEG segments into three clinically relevant categories: Normal, Slow Waves (SW), or Spike and Sharp Waves (SSW). Our pipeline detects individual events within EEG recordings, allowing decisions based on individual events within recordings instead of black-box style, classification decisions based on the whole recording.
- We examine the computational efficiency and classification performance of feature extraction pipelines based on Fourier and Wavelet features and benchmark them against a pipeline based on raw time samples. This enables us to quantify the performance gains (if any) and the increase in computational overhead associated with these feature extraction techniques.
- LEEDNet is evaluated on the NMT-Events dataset, in which EEG events are annotated by experts with high temporal resolution. Given the small temporal span of the target events, the length of the classification window also has a significant impact on the computational overhead. We vary the window/segment length to determine which value demonstrates the correct balance between performance and computational efficiency.
- Our analysis provides valuable insights for field deployments in resource-constrained settings and is released as open-source.^1^

## Related work

**Fig. 1:**
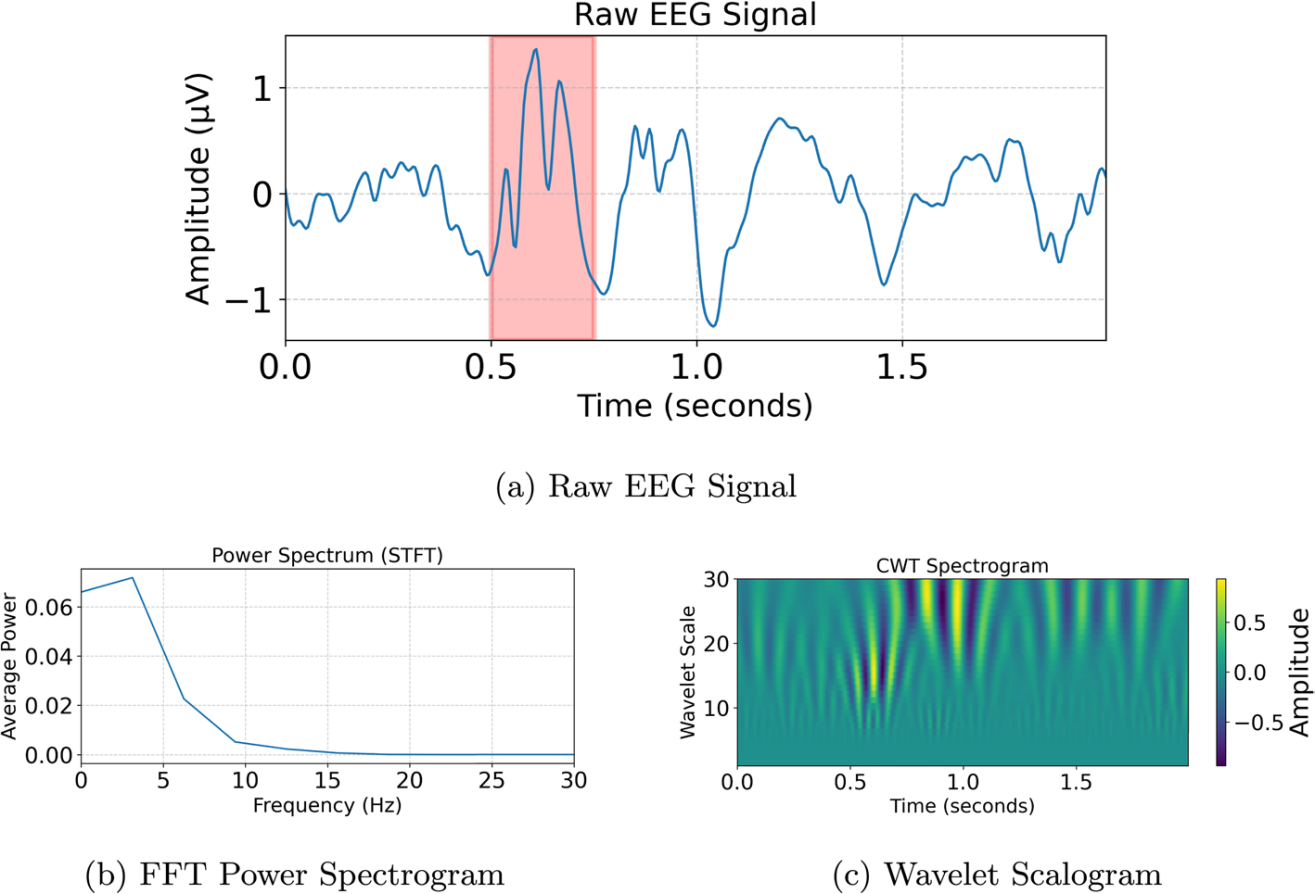
Examples of EEG segment representations used in this study. **a** Time-domain EEG retains raw waveform structure, allowing detection of morphological transients along with the label indicating the event location. The red-shaded region indicates the exact temporal span of the spike-and-wave event. **b** FFT-based spectrogram highlights frequency-domain rhythms. **c** Morlet-based wavelet scalogram captures localized transient time–frequency activity

Automated EEG event detection and localization have been studied using traditional machine learning, deep learning, and, more recently, transformer-based models. Early approaches relied on handcrafted features extracted from the time, frequency, or time–frequency domains—such as wavelet energy, entropy, or spectral power [6]. These features were typically fed into classifiers like SVMs [7], decision trees [8], or random forests. While interpretable, such pipelines require expert tuning and often generalize poorly. To overcome these limitations, some recent approaches have employed graphical features in conjunction with binary particle swarm optimization [16, 17]. After the emergence of deep learning, models such as EEGNet [9], ChronoNet [10], HybridDeepCNN [18], and DeepConvNet [11] demonstrated that spatiotemporal patterns can be learned directly from raw EEG signals. These models have been widely applied to seizure detection, sleep staging, and BCI classification. However, these studies classify a whole EEG recording as normal or pathological, which is acceptable for screening purposes but not for triage applications, where more information regarding events within the EEG recording is needed to support patient prioritization and assessment. For improved quantification, segment-level EEG classification has emerged. Golmohammadi et al. [19] applied speech-derived features, such as MFCCs and LFCCs, to classify six types of EEG events. Although innovative, these features were originally designed for speech recognition and do not generalize well to the EEG domain. NeuroAssist [14] advanced this direction by introducing the NMT-Events dataset, which contains short EEG segments labeled by experts as slow waves (SW) and spike and sharp waves (SSW); a sample (single-channel) EEG signal, along with the corresponding event label, is shown in Fig. 1a. Their wavelet-CNNs approach relied on continuous wavelet transform (CWT)-based scalograms and 2D CNNs such as VGG16. While effective, this pipeline is not computationally efficient due to its reliance on large-parameter deep learning architectures and a CWT-based feature extraction block. Some other related works include [20] and [21] which employ signal denoising and decomposition approaches to develop robust motor imagery classification approaches. Along the same lines, some researchers have employed CNNs in conjunction with phase space features [22] and CWT features [23]. However, these approaches mostly focus on BCI applications, whereas our work is primarily concerned with detection of pathological events within clinical EEG data.

Recent work has explored transformer-based models for EEG. EEGformer [24] and EEG-Deformer [25] applied self-attention to model EEG sequences for emotion and motor decoding. ScatterFormer [26] used scattering transforms and transformers to detect epileptiform discharges in a patient-independent setting. EEGPT [27] introduced a contrastive-pretrained transformer encoder capable of transferring across tasks, such as seizure detection and sleep staging. EENED [28] combined CNNs and transformers to detect epileptic events with strong performance. A transformer-GRU fusion for seizure prediction was presented by Zhu et al. [29], achieving high sensitivity on the CHB-MIT dataset. However, transformer-based models incur substantially higher computational overhead and must be assessed from this perspective before deployment in resource-constrained environments. Some very recent works in [30–32], and [33] have examined lightweight approaches for classification of EEG data but again are more focused on BCI applications instead of detection pathological EEG events. In [33], a Spatial Group-wise Enhance (SGE) module is coupled with CNNs to develop a lightweight architecture for motor imagery classification; however, performance has not been examined in terms of latency or FLOPs. Similarly, in [32] computational efficiency has been identified as one of the contributions but has not been examined extensively in the results. In [31], inference time and model parameters have been presented but only on existing architectures with no new architecture being proposed. A novel lightweight architecture, based on spiking neural networks, has been proposed for motor imagery classification in [30] but latency and inference time have not been provided.

Our work addresses the gaps in the existing literature by introducing LEEDNet, a lightweight model that classifies short, single-channel EEG windows into Normal, Slow Waves (SW), and Spike and Sharp Waves (SSW). In addition to conventional performance evaluation metrics, we also assess the computational overhead associated with LEEDNet and benchmark it against the latest existing architectures in terms of latency, number of parameters, and processing speed (FLOPs) of standalone CPU and CPU+GPU deployments. Our analysis will be useful for researchers interested in developing automated solutions for EEG triage applications in resource-constrained environments.

## Methodology

This section describes the complete methodological pipeline developed for segment-level EEG event classification. It includes dataset preparation, preprocessing, model architecture design (both baseline and advanced), training strategies, and evaluation metrics, all supported by evidence from clinical EEG practice and machine learning research. The approach prioritizes low-latency processing and minimal preprocessing for effective deployment in hospital and portable monitoring environments.

### Dataset and segment annotation

We evaluate our approach on the publicly available NMT-Events dataset [14], comprising 1,075 clinical EEG recordings acquired, from distinct subjects/participants, at 200 Hz using the international 10–20 system (21 channels). The recordings were manually annotated by board-certified neurologists, identifying two key classes of pathological EEG events: Slow Waves (SW) and Spike and Sharp Waves (SSW). Segments that do not overlap with abnormal annotations are labeled as Normal. Overall, nine different categories of events are available in the NMT-Events dataset. However, for the purpose of this work these nine sub-events were merged into the two broad event labels (SW and SSW) as follows: SW subsumes sharp and slow wave, delta slow wave, and sharp and delta slow waves. SSW subsumes spike and wave, spikes, polyspikes, polyspikes and wave, and sharp wave. Following the evaluation protocol in [14], for the purpose of event localization, each EEG recording was segmented into overlapping windows of 2 s, with a 50% overlap between consecutive segments. Each window was annotated with one of three event categories according to the neurologists’ reference label files. A short window duration was deliberately chosen to ensure fine temporal precision. Nevertheless, this segmentation strategy introduces a discretization effect, as windows located at event boundaries may only partially capture an event. To mitigate this issue, a discretization criterion of 25% was applied: any window overlapping an event (SW or SSW) by at least 25% was labeled as an event window and treated as if the event spanned the full window duration. Due to the pronounced class imbalance in the dataset—where normal activity substantially exceeded abnormal events—class balancing was performed to reduce classifier bias. Specifically, a random subset of normal windows was selected to approximately equal the number of abnormal windows (combining SW and SSW). The selection of normal samples followed a two-step procedure: (i) randomly sampling normal windows from recordings without any abnormal events, and (ii) extracting fully normal windows from recordings that contained abnormalities. The resulting dataset was divided into *training*, *validation*, and *test* sets using a subject-based split rather than a window-level random split. This means that all data windows from a given participant/subject were assigned exclusively to a single subset (training, validation, or test), thereby preventing data leakage across partitions. Details regarding subset sizes and class distributions are provided in Table 1.

**Table 1:** Dataset partitioning into training, validation, and testing sets, along with the distribution of window labels for each set

Dataset partition	Number of recordings	Label distribution		
		Normal	SW	SSW
Train	726	149,025	94,965	63,085
Valid	135	25,540	16,758	11,132
Test	214	36,563	25,039	13,172

The labels include Normal, Slow Waves (SW), and Spike and Sharp Waves (SSW) conditions

### Preprocessing

All EEG recordings are resampled (if needed) to a unified sampling rate of 200 Hz to ensure temporal consistency across subjects. We then apply a Hamming-windowed FIR band-pass filter with a pass band of 1–45 Hz, effectively removing DC drift, movement artifacts, and muscle noise while preserving frequencies critical for clinical interpretation [3, 34]. To suppress non-cerebral artifacts, we apply Independent Component Analysis (ICA), a widely used method for isolating and removing stereotypical artifacts such as eye blinks, cardiac signals, and muscle contractions [35]. These steps ensure that the input signals reflect true neural activity while minimizing non-neural interference. Multiscale Principal Component Analysis (MSPCA) is another popular denoising technique for EEG data. It has been used successfully to develop robust algorithms for classification of BCI data in [36] and [37]. As an alternative to ICA, we implemented MSPCA at the preprocessing stage, in accordance with the guideline provide in [37]; however, the performance observed for our dataset was not better than ICA.

Each extracted EEG segment is represented as a matrix of shape C×T. This work focuses on a single-channel representation that is consistent with the annotation information in the NMT-Events dataset. Consequently, C=1 results in a vector of length $T = \textit{No. of time samples in one segment}$. The resulting set of segment vectors is then normalized to zero mean and unit variance per recording to reduce inter-subject variability and improve numerical stability during training.

### Input modalities

To evaluate the contribution of different feature extraction approaches, we constructed three complementary input modalities, as depicted in Fig. 1, which were later used both independently and in fusion:

- *Raw EEG (time domain):* It preserves the original waveform structure, allowing the model to learn directly from morphology such as spikes, sharp waves, and slowing, which are critical for detecting epileptiform and other abnormal activities.
- *FFT-based spectra:* The Short-Time Fourier Transform (STFT) was applied to band-pass filtered EEG signals (1–45 Hz) to generate power spectrograms that emphasize rhythmic and spectral dynamics indicative of pathological slowing.
- *Wavelet scalograms:* The Continuous Wavelet Transform (CWT) using a Morlet mother wavelet produces localized time–frequency representations. These retain both spectral and temporal details, capturing transient oscillatory events such as spike–wave or polyspike bursts.

Each modality-specific input is formatted as a tensor of shape [*B*, *C*, *H*, *W*], where *B* is the batch size, *C* is the number of channels (typically 1), and H×W represents the modality-specific dimensions (e.g., temporal length for raw data or frequency–time grid for FFT and wavelet analysis). All inputs are independently normalized before being passed into LEEDNet’s single- or multi-branch architectures.

### Model architecture

**Fig. 2:**
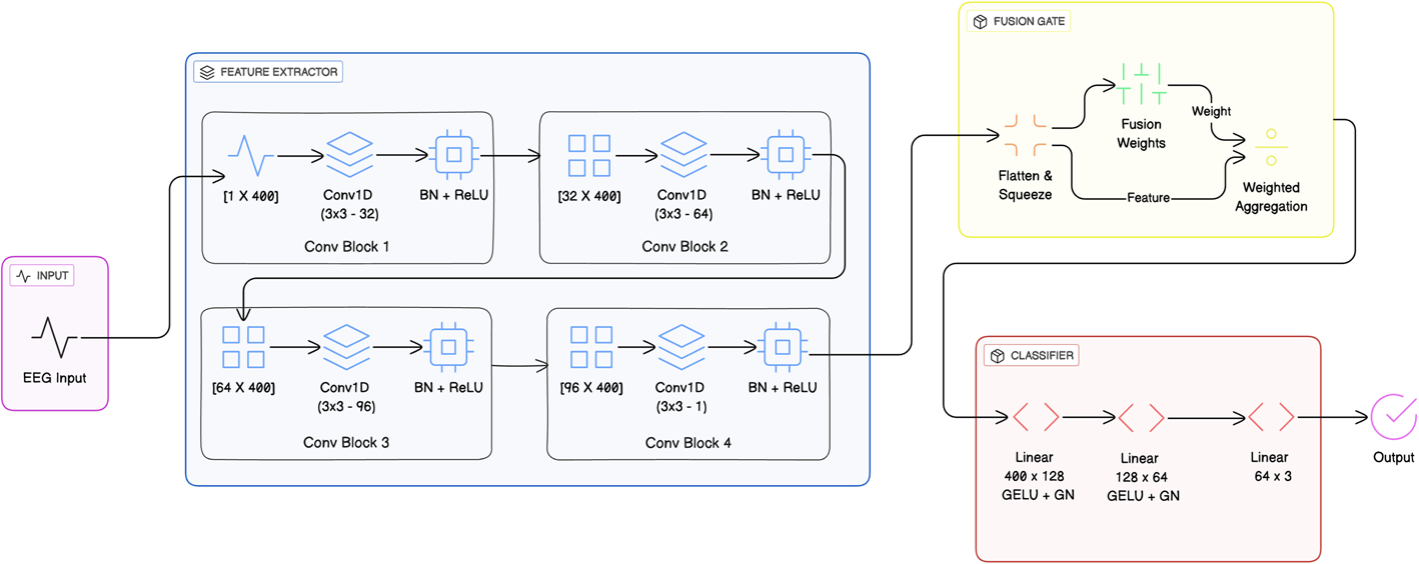
Detailed architecture of single-branch LEEDNet. Temporal features are extracted using 1D convolution blocks with increasing filter depths, followed by a fusion gate for temporal attention and a classifier for event prediction. This architecture was independently trained with raw EEG, FFT, and wavelet inputs to evaluate modality-specific performance

**Fig. 3:**
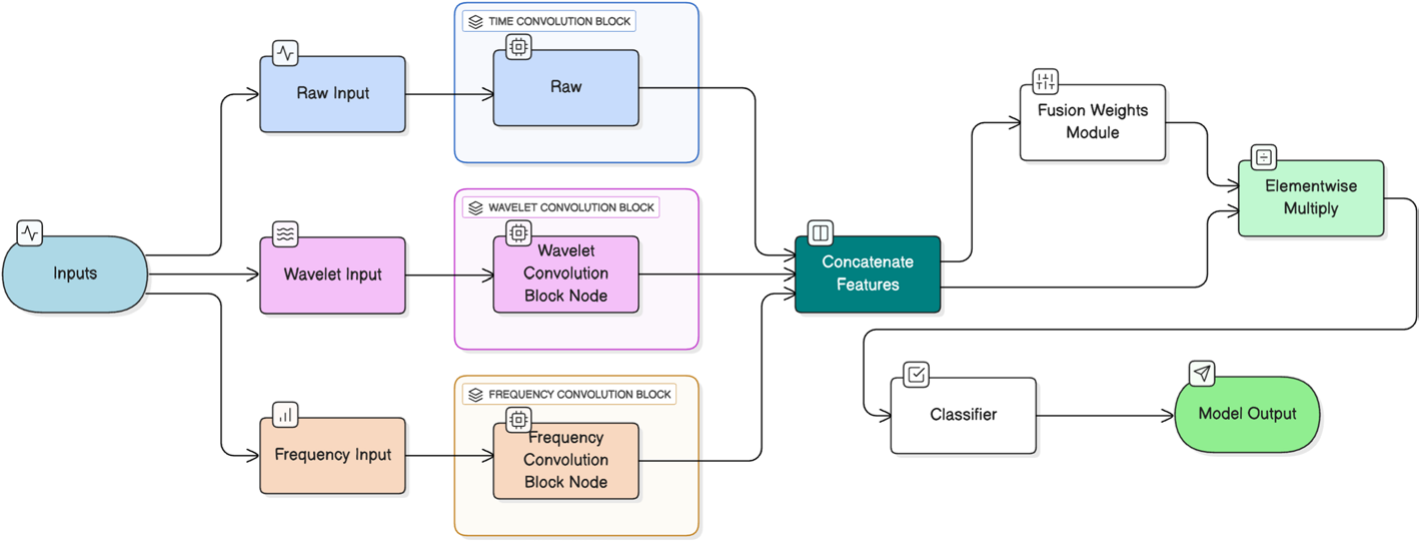
Multi-branch LEEDNet architecture. The model processes raw, wavelet, and frequency-domain inputs via separate convolutional blocks. Extracted features are concatenated and reweighted using a Fusion Weights Module before classification. This architecture allows flexible evaluation of single or combined modalities for EEG event detection

We propose LEEDNet (Lightweight EEG Event Detection Network), a lightweight convolution–attention framework optimized for clinical EEG analysis. It achieves high efficiency (<0.5 M parameters; <1 ms inference per 2-second segment on GPU) while maintaining real-time CPU performance. LEEDNet’s design follows two principles: lightweight temporal attention and computational scalability. Two complementary configurations were developed: (1) a single-branch variant for modality-specific evaluation, and (2) a multi-branch variant that fuses multiple EEG representations. Their structures are shown in Figs. 2 and 3.

#### Single-branch LEEDNet

The single-branch LEEDNet is a lightweight and flexible architecture designed to process one EEG modality at a time—either raw signals, FFT-derived spectrograms, or wavelet-based scalograms. It comprises three primary components: a feature extraction module, an attention-driven fusion gate, and a classification head. In this study, we independently trained and evaluated the single-branch LEEDNet on each of the three input modalities to systematically examine their individual contributions to EEG event classification. This design enabled a fair comparison of the discriminative power of each representation within a consistent neural framework.

Module M1: feature extractor (FE)

The Feature Extractor (FE) is responsible for learning localized temporal patterns from short EEG segments that are diagnostically relevant, such as sharp transients, spike discharges, and rhythmic slowing. Each EEG segment is represented as a one-dimensional tensor $\textbf{x} \in \mathbb {R}^{B \times C \times T}$, where *B* denotes the batch size, *C* is the number of input channels (typically 1), and *T* corresponds to the number of time samples in the input EEG segment/window. As illustrated in Fig. 2, the FE consists of four sequential one-dimensional convolutional blocks that progressively refine temporal representations while preserving temporal resolution. The first three convolutional blocks increase channel depth from 32 to 96, enabling hierarchical feature abstraction in line with established deep CNN design principles [38, 39]. Formally, the operations are defined as:

$$\begin{aligned} h_1&= \phi \!\big (\textrm{BN}(\textrm{Conv1D}_{C \rightarrow 32}^{k=3,s=1}(x))\big ),\\ h_2&= \phi \!\big (\textrm{BN}(\textrm{Conv1D}_{32 \rightarrow 64}^{k=3,s=1}(h_1))\big ),\\ h_3&= \phi \!\big (\textrm{BN}(\textrm{Conv1D}_{64 \rightarrow 96}^{k=3,s=1}(h_2))\big ), \end{aligned}$$

where ϕ denotes the GELU activation, *k* is the kernel size, and *s* is the stride. Each convolution uses “same” padding to maintain the temporal length *T*, followed by Batch Normalization to stabilize learning and accelerate convergence.

The first two layers, with 3-point receptive fields and 32–64 filters, capture high-frequency transients such as spikes and sharp waves, while the progressive increase in channel depth enhances representational capacity for learning more complex patterns. The third layer, with 96 filters, models broader and slower temporal structures, including rhythmic or compound discharges. Together, these stages enable the network to learn a hierarchy of temporal features spanning different EEG event durations.

Finally, a projection layer compresses the learned feature maps into a single-channel representation:

$$\tilde{h} = \textrm{BN}\!\big (\textrm{Conv1D}_{96 \rightarrow 1}^{k=3,s=1} (h_3)\big ),$$

producing an output tensor $\tilde{h} \in \mathbb {R}^{B \times 1 \times T}$ that retains essential waveform morphology while reducing feature dimensionality. This morphology-preserving embedding is subsequently passed to the Temporal Fusion Gate for attention-based weighting.

To achieve computational efficiency suitable for real-time deployment, the FE employs depthwise–separable convolutions, which reduce parameter count and floating-point operations without sacrificing representational capacity. This design approach, inspired by lightweight architectures such as InceptionTime [40], enables LEEDNet to perform efficient inference on low-resource clinical and portable hardware. By avoiding temporal pooling and complex transformations, the FE preserves waveform integrity and alignment—mirroring the visual inspection process of clinical EEG interpretation [3]—while maintaining high processing throughput for real-time event detection [5].

Module M2: fusion gate (FG)

The Fusion Gate (FG) serves as a temporal attention mechanism that enables the model to emphasize diagnostically relevant portions of the EEG signal while suppressing less informative regions. It operates on the output of the Feature Extractor, $\tilde{h} \in \mathbb {R}^{B \times 1 \times T}$, which is first flattened along the temporal axis to form a two-dimensional representation:

$$u={\textrm{vec }}(\tilde{h}) \in \mathbb {R}^{B \times T}.$$

This flattened representation is passed through a two-layer fully connected network composed of a dimensionality reduction layer followed by a projection layer:

$$\alpha = \sigma \!\big (W_2\,\textrm{ReLU}(W_1 u)\big ),$$

where $W_1 \in \mathbb {R}^{(T/2)\times T}$ performs dimensionality reduction, $W_2 \in \mathbb {R}^{T\times (T/2)}$ projects the features back to their original size, $\textrm{ReLU}$ introduces nonlinearity, and σ denotes the Sigmoid activation. The resulting attention weights $\alpha \in [0,1]^T$ represent the relative importance of each time point in the segment.

These learned weights are applied element-wise to the temporal feature vector:

$$g = \alpha \odot u,$$

where ⊙ denotes element-wise multiplication. The resulting gated signal $g \in \mathbb {R}^{B \times T}$ retains the original temporal resolution while emphasizing salient waveform regions and attenuating irrelevant activity. This mechanism enhances the model’s ability to focus on clinically meaningful temporal structures, improving its discriminative capacity without increasing computational complexity.

Module M3: classifier head

The Classifier Head is a shallow multilayer perceptron (MLP) that maps the temporally gated representation *g* to logits for three EEG event classes: $\{Normal, SW, SSW\}$. Let the input dimensionality be *T* (i.e., the temporal width of *g*). The transformation is

$$\begin{aligned} g \rightarrow \;&\textrm{FC}(T \!\rightarrow \! 128) \rightarrow \textrm{GELU} \rightarrow \textrm{GN} \rightarrow \textrm{Drop}(0.4)\\&\rightarrow \textrm{FC}(128 \!\rightarrow \! 64) \rightarrow \textrm{GELU} \rightarrow \textrm{Drop}(0.2) \rightarrow \textrm{FC}(64 \!\rightarrow \! 3) \rightarrow \textrm{Softmax}, \end{aligned}$$

Here, FC denotes a fully connected (linear) layer, GELU the Gaussian Error Linear Unit nonlinearity, GN Group Normalization, and $\textrm{Drop}(p)$ standard dropout with a drop probability of *p*. Group Normalization is employed instead of Batch Normalization to ensure stable training under varying batch sizes. This shallow classifier balances expressiveness and generalization, allowing the primary discriminative power to originate from the preceding convolutional and attention modules.

#### Multi-branch LEEDNet

To investigate whether integrating heterogeneous EEG representations can improve event classification performance, we designed a modular multi-branch variant of LEEDNet (Fig. 3). This architecture concurrently processes three complementary input modalities—raw EEG signals, FFT-based spectral features, and wavelet-derived time–frequency representations—through parallel feature extraction pathways. Each branch follows the same convolutional and attention backbone as the single-branch LEEDNet but maintains independent parameters, allowing modality-specific feature adaptation. The modality-specific embeddings are subsequently fused through an adaptive weighting mechanism and passed to a shared classifier for final prediction.

Module B0: parallel encoders

The Parallel Encoders form the foundational component of the multi-branch LEEDNet, enabling modality-specific feature extraction across three complementary EEG representations: raw time-domain signals, FFT-based spectral features, and wavelet-based time–frequency scalograms. Each input modality is processed independently through its own encoder branch, structurally identical to the Feature Extractor and Fusion Gate modules used in the single-branch LEEDNet (Modules M1–M2 in Fig. 2). Although the architectural design is shared, each branch maintains independent trainable parameters, allowing adaptation to the unique characteristics of its respective modality.

For STFT and CWT inputs, an initial $1{\times }1$ temporal projection layer flattens channelized spectral features into a single temporal stream before convolutional processing. Each encoder branch outputs a gated temporal embedding $g^{(m)} \in \mathbb {R}^{B \times T}$, where $m \in \{\text {raw}, \text {fft}, \text {cwt}\}$ denotes the modality. These embeddings are subsequently fused through an adaptive weighting mechanism in the Fusion Weights Module.

Module B1: fusion weights module (FWM)

The Fusion Weights Module (FWM) integrates information from multiple encoder branches by extending the attention-based weighting mechanism introduced in the single-branch LEEDNet (see Section 3.4.1). The modality-specific embeddings $g^{(m)}$ from the raw, FFT, and CWT encoders are concatenated along the feature dimension to form a unified representation $\textbf{z} \in \mathbb {R}^{B \times (3T)}$ for tri-modal fusion (or 2*T* for bi-modal configurations).

The same two-layer multilayer perceptron (MLP) formulation defined for the Fusion Gate (FG) is reused here to learn adaptive weights across modalities:

$$\mathbf {z'} = \textbf{z} \odot \sigma (W_2^{(f)} \, \textrm{ReLU}(W_1^{(f)} \textbf{z})),$$

where $W_1^{(f)}$ and $W_2^{(f)}$ are modality-specific weight matrices that are reinitialized for fusion, σ denotes the Sigmoid activation, and ⊙ represents element-wise multiplication. The MLP reduces and then restores the feature dimensionality, allowing the network to learn channel-wise attention weights that highlight the most informative modality subspaces.

This adaptive reweighting mechanism allows LEEDNet to automatically prioritize discriminative modality features while suppressing less informative or noisy ones, thereby enhancing both robustness and interpretability in multi-modal EEG fusion.

Module B2: classifier

The weighted feature vector $\mathbf {z'}$ is passed through a three-layer classification head, identical to the one used in the single-branch LEEDNet. It maps the features to class logits, which are converted to probabilities via Softmax activation, producing the final event prediction: Normal, SW, or SSW.

Modality configurations

To systematically evaluate the contribution of each EEG representation and their combinations, the multi-branch LEEDNet was assessed under multiple modality configurations. Each configuration selectively activated a subset of the available encoder branches—Raw, FFT, and Wavelet—while keeping the overall architecture, including the Fusion Weights Module (FWM) and classifier, unchanged. This design ensured consistent parameterization and normalization behavior across experiments, isolating the impact of each modality or modality pair.

The following input combinations were evaluated:

1. **Raw + FFT + Wavelet:** All three branches were active, enabling comprehensive multi-modal feature extraction and fusion.
2. **Raw + FFT:** Combined time-domain and frequency-domain representations, excluding wavelet-based inputs.
3. **Raw + Wavelet:** Integrated temporal and time–frequency information while omitting FFT-based features.
4. **FFT + Wavelet:** Captured complementary spectral and time–frequency dynamics, excluding raw-signal input.

In each configuration, inactive branches were omitted from concatenation within the FWM to maintain consistent normalization and attention statistics. This systematic evaluation allowed us to quantify the relative contribution of each modality and to determine whether multi-modal fusion yielded tangible performance gains over single-modality processing.

The parallel encoder design enables modality-specific specialization, while the Fusion Weights Module performs efficient cross-modal recalibration—conceptually similar to the squeeze-and-excitation mechanism [41]—but with significantly lower latency and without the computational overhead of full self-attention.

### Training protocol

All models were trained using the AdamW optimizer with parameters $\beta _1 = 0.9$, $\beta _2 = 0.98$, $\epsilon = 1 \times 10^{-9}$, and a weight decay of $1 \times 10^{-4}$. The initial learning rate was set to $1 \times 10^{-4}$, and a ReduceLROnPlateau scheduler was employed to adjust the learning rate dynamically. Specifically, the scheduler reduced the learning rate by a factor of 0.5 if the validation loss did not improve for 3 consecutive epochs.

The loss function used was cross-entropy with label smoothing ($\epsilon _{ls} = 0.1$), which encourages the model to produce less confident predictions and improves generalization, especially in the presence of class imbalance. Additionally, inverse class weighting was applied to the loss function, ensuring that underrepresented classes (e.g., Slow Waves (SW) and Spike and Sharp Waves (SSW)) were emphasized appropriately during training.

Training was conducted using a batch size of 64, with early stopping based on validation loss (patience = 10 epochs) to prevent overfitting. Models were trained for a maximum of 100 epochs; however, early stopping typically triggered earlier convergence. To ensure robust generalization and avoid data leakage, we employed a subject-wise data partitioning strategy. All EEG segments from a given subject were assigned exclusively to one of the training, validation, or test sets. This ensures that no subject appears in more than one subset, enabling evaluation on completely unseen subjects. All results reported in Tables 2, 3 and 4 are obtained using this fixed subject-wise split.

All experiments were conducted on a high-performance workstation equipped with an NVIDIA RTX 3090 GPU (24 GB VRAM), an Intel Core i-9 processor, and 64 GB of RAM, running PyTorch 1.13 with CUDA acceleration. This setup allowed for efficient training and evaluation of both the single-branch and multi-branch LEEDNet architectures.

### Evaluation metrics

To comprehensively evaluate the performance of LEEDNet and its variants, we report both diagnostic accuracy metrics and computational efficiency indicators. All evaluations were conducted on held-out test sets using a subject-wise data split, such that all EEG windows belonging to a given subject were assigned exclusively to either the training, validation, or testing set.

#### Diagnostic performance

We adopt four complementary metrics to assess classification effectiveness:

- *Accuracy:* The percentage of EEG segments correctly classified into one of the three categories—Normal, SW, or SSW. Although accuracy offers an overall view of performance, it may be influenced by class imbalance and is therefore supplemented by class-sensitive measures.
- *Macro-F1-Score:* The harmonic mean of precision and recall, computed independently for each class and averaged across all classes. This metric provides a balanced assessment, especially when the accurate detection of both SW and SSW events is clinically critical.
- *Sensitivity (Recall):* The proportion of true abnormal segments (SW and SSW) that are correctly identified. High sensitivity minimizes the risk of missed pathological events—an essential property for clinical EEG interpretation.
- *Specificity:* The proportion of Normal EEG segments correctly identified. High specificity ensures that benign EEG activity is not misclassified as abnormal, reducing false positives and improving clinical reliability.

Given the diagnostic relevance of detecting abnormal EEG patterns, we emphasize macro-F1, class-wise recall (sensitivity), and specificity as primary performance indicators, with accuracy serving as a complementary global measure.

#### Computational efficiency

In addition to classification metrics, we evaluate the computational footprint of each model configuration to assess its suitability for real-time and portable deployment. The following metrics are reported:

- *Parameters:* Total number of trainable parameters indicating model compactness.
- *FLOPs:* Estimated floating-point operations per forward pass (computed for $T{=}400$ samples), representing the computational cost.
- *Latency:* Average wall-clock inference time per 2-second EEG segment, measured on both GPU and CPU (batch size 1). Lower latency indicates higher real-time feasibility.

These efficiency metrics provide a quantitative view of LEEDNet’s resource footprint across different modality configurations (single and multi-branch). By jointly evaluating diagnostic accuracy and computational cost, we ensure that LEEDNet achieves an optimal balance between clinical performance, interpretability, and deployability in real-time hospital and portable EEG monitoring settings.

## Results and discussion

This section presents the empirical evaluation of LEEDNet across multiple experimental dimensions, including input modality, segment length, and learned feature representations. Comparative analysis with state-of-the-art models and interpretability visualizations is also provided.

**Table 2:** Comparison of single-branch LEEDNet for features based on different types of signal decomposition techniques

Representation	Sensitivity **(%)**	Specificity **(%)**	Accuracy **(%)**	F1-score **(%)**
*Continuous wavelet transform (CWT)*				
LEEDNet (Complex Morlet)	76.00	**89**.**24**	**78**.**12**	**74**.**87**
LEEDNet (Morlet)	71.42	87.63	75.11	70.45
LEEDNet (Mexican Hat)	**76**.**72**	89.12	78.02	74.72
LEEDNet (Gaussian, gaus5)	74.08	88.28	76.68	72.98
*Discrete wavelet transform (DWT)*				
LEEDNet (Symlet)	73.99	88.08	75.79	72.43
LEEDNet (Daubechies, db4)	73.74	88.08	75.88	72.24
*Variational mode decomposition (VMD)*				
LEEDNet (VMD)	73.67	87.65	75.40	72.57
LEEDNet (VMD + Statistical Features)	50.07	79.60	62.83	47.32
LEEDNet (VMD + Complex Morlet)	74.19	88.49	76.78	73.02

### Feature extraction using signal decomposition techniques

A wide variety of wavelet techniques is available for processing of EEG signals. To select an appropriate wavelet transform for our target task we assessed the performance of features extracted using different mother wavelets and then feeding these into the single-branch LEEDNet model. The performance metrics observed on the test dataset are presented in Table 2. It can be observed that the Complex Morlet delivers the best performance in terms of Specificity, Accuracy and F1-score therefore, we used this wavelet for all subsequent experiments with wavelets. In addition to wavelets, a number of other signal decomposition techniques are also available for EEG feature extraction. One of the most popular options is variational mode decomposition (VMD) [42] and it multivariate extension, multivariate variational mode decomposition (MVMD) [43], which has been used for motor imagery classification in BCI applications with high accuracy in [37] and [44]. LEEDNet operates on one EEG channel at one time; therefore, VMD was used to decompose an input EEG window into 5 Intrinsic Mode Functions (IMFs), this resulted in $5\times 400(=\text {No. of time samples})$ raw features which were then used to train our model. This approach resulted in an F1-score of 72.57%. We also implemented another variant of VMD, according to the guidelines provided in [44]. For this approach, we extracted features representing the spectral and statistical characteristics of each IMF. These include: (1) Energy: The sum of squares of the coefficients, representing the total power of the mode; (2) Statistical Features: mean, variance, standard deviation, kurtosis and skewness; (3) Shanon Entropy; (4) Frequency-Domain Features: spectral centroid, bandwidth, and peak frequency (the frequency at which the maximum power occurs). Contrary to our expectations, the model trained using these features did not deliver a high performance. Finally, VMD was also used in conjunction with wavelet features. Given that the Complex Morlet was the best performing mother wavelet, we applied it to output of VMD to construct features. In particular, a Complex Morlet with 30 scales (using the cmor1.5−1.0 wavelet) was calculated for each of the 5 IMFs. The 5 resulting time–frequency maps (each (30×400)) were then averaged together. This produced a single VMD-Enhanced Time–Frequency Map of shape (30×400), which was fed into the LEEDNet model for training and testing. This approach improved performance compared to the VMD on raw IMFs and the VMD + Statistical Features approaches; however, it was unable to outperform the features based on only the Complex Morlet transform. Consequently, we decided to use only the best performing decomposition (Complex Morlet) as the wavelet input for all subsequent experiments.

**Table 3:** Comparison of different LEEDNet configurations with prominent baselines on 2-second EEG segments from *NMT-Events*

**Model**	**Sensitivity** **(%)**	**Specificity** **(%)**	**Accuracy** **(%)**	**F1-score** **(%)**
InceptionTime [40]$^{\ddagger }$	78.56	90.61	79.91	76.03
MiniRocket [45]$^{\ddagger }$	76.20	90.11	80.42	76.74
GoogLeNet [14]	75.40	87.01	78.83	74.20
VGG16 [14]	76.70	89.60	79.20	74.60
EEGConformer [46]$^{\ddagger }$	**79**.**32**	89.89	77.95	75.18
EEGNet [9]$^{\ddagger }$	73.80	87.58	72.06	68.98
LEEDNet (FFT)	69.75	87.65	73.01	68.47
LEEDNet (Wavelet)	76.00	89.24	78.12	74.87
LEEDNet (Raw+FFT)	77.08	89.74	80.41	76.49
LEEDNet (Raw+Wavelet)	72.64	88.56	76.64	71.93
LEEDNet (FFT+Wavelet)	72.22	86.91	75.83	71.28
LEEDNet (Raw+FFT+Wavelet)	73.01	88.48	76.60	72.10
**LEEDNet (Raw)**	78.07	**90**.**76**	**81**.**22**	**77**.**49**

The wavelet used here is the Complex Morlet. Metrics reported are Sensitivity, Specificity, Accuracy, and F1-score. Entries marked with $^{\ddagger }$ are our re-evaluations on *NMT-Events* (using released code for faithful re-implementations with standardized preprocessing and subject-wise splits) because the original papers did not report results on this dataset

### Performance comparison across modalities and baselines

Table 3 presents a detailed comparison of LEEDNet’s performance across six different modality configurations. These configurations are further benchmarked against a set of competitive state-of-the-art (SOTA) models frequently used in EEG classification tasks.

Among all LEEDNet configurations, the single-branch LEEDNet (Raw) achieved the best overall performance, with a sensitivity of 78.07%, specificity of 90.76%, accuracy of 81.2%, and F1-score of 77.49%. These results underscore the value of modeling EEG in the time domain using raw, untransformed signals, which retain critical waveform morphology necessary for identifying clinically significant patterns such as spike discharges, sharp waves, and slowing events. The raw-only LEEDNet, despite its architectural simplicity, proved to be both robust and generalizable across test subjects.

Multi-modal LEEDNet variants, which integrate frequency (FFT) and time–frequency (wavelet) features, did not yield significant performance gains. For instance, the dual-branch LEEDNet (Raw+FFT) achieved slightly lower accuracy (80.4%) and F1-score (76.49%) than the raw-only model, while the tri-modal LEEDNet (Raw+FFT+Wavelet) further reduced the accuracy and F1-score to 76.6% and 72.10% respectively. These findings suggest that while additional modalities may offer complementary information, they can also introduce quantization noise, redundancy, and increased model complexity, leading to the overfitting of particular classes, particularly in datasets with moderate class imbalance and limited training samples.

Single-modality models using only FFT or wavelet features generally underperformed relative to LEEDNet (Raw). LEEDNet (FFT) achieved 73.01% accuracy, while LEEDNet (Wavelet), using the Complex Morlet representation, achieved 78.12% accuracy. Although the wavelet-based model performed better than the FFT-only variant, it still remained below the raw-signal model. Their lower sensitivity and F1-scores indicate a reduced ability to detect transient, clinically relevant EEG abnormalities. These results seem to support the hypothesis that time-domain features derived directly from raw EEG are more informative and discriminative for segment-wise abnormality detection.

LEEDNet (Raw) not only outperformed all other LEEDNet configurations but also delivered highly competitive results when compared to state-of-the-art (SOTA) models. Specifically, it surpassed EEGNet [9], a compact CNN designed for EEG-based brain–computer interfaces, which achieved 72.06% accuracy and a 68.98% F1-score. It also exceeded the performance of InceptionTime [40], a temporal CNN leveraging inception modules, which obtained 79.91% accuracy and a 76.03% F1-score. Furthermore, LEEDNet (Raw) outperformed the wavelet-CNN pipeline developed by Alqarni et al. [14] for clinical EEG event localization, which reached 79.2% accuracy and a 74.60% F1-score.

While MiniRocket [45] achieved slightly higher accuracy (80.42%) than several LEEDNet multi-modal variants, it fell short of the single-branch LEEDNet (Raw) in both accuracy (80.4% vs. 81.2%) and macro-F1-score (76.74% vs. 77.49%). This indicates a less balanced performance across classes. In contrast, LEEDNet (Raw) offers a lighter, more interpretable architecture with temporal attention visualization, ensuring both efficiency and explainability in EEG-based diagnosis.

The transformer-based model EEGConformer [46] achieved the highest sensitivity (79.32%) among all models, with 77.95% accuracy and a 75.18% F1-score. Its specificity (89.89%) was comparable to LEEDNet (Raw); however, these differences are offset by higher latency associated with EEGConformer model as demonstrated in the next section.

From a clinical perspective, the high specificity of LEEDNet (Raw) (90.76%) is particularly valuable, as it reduces false positives (misclassifying normal EEG as abnormal), which can lead to unnecessary diagnostic follow-ups or interventions. At the same time, its strong sensitivity (78.07%) ensures that the majority of abnormal events (Slow Waves (SW) and Spike and Sharp Waves (SSW)) are correctly identified, minimizing the risk of under-diagnosis.

### Computational efficiency

The most compact variant, LEEDNet (Raw), contains 0.253 M trainable parameters and requires approximately 37 MFLOPs per segment. It attains an average inference time of 0.86 ms on the CPU and 0.85 ms on the GPU (Table 4). This compact footprint enables real-time operation on edge devices without specialized accelerators.

For context on the same task, GoogLeNet [14] uses 8.48M parameters and 3.01 GFLOPs per segment, i.e., ∼33.5× more parameters and ∼81.4× higher compute than LEEDNet (Raw), with a measured latency of 60 ms (CPU) and 36.4 ms (GPU), about ∼70× and ∼43× slower, respectively. VGG16 is heavier at 138.36M parameters and 30.96 GFLOPs (∼547× parameters, ∼837× compute), with 250 ms (CPU) and 43 ms (GPU)–approximately ∼291× and ∼51× slower than LEEDNet (Raw). Among our variants, adding frequency branches increases computation modestly but remains within real-time limits: for example, LEEDNet (Raw+FFT+Wavelet) has 0.768M / 120 MFLOPs with 3.19 ms (CPU) / 2.53 ms (GPU), while the single-branch LEEDNet (FFT) and LEEDNet (Wavelet) are typically 1–2 ms on both CPU and GPU.

We also evaluated EEGConformer with the same input (1 × 400), which activates a reduced subgraph and, therefore, reports a small footprint (0.34M parameters, 7.51 MFLOPs). Despite this lower nominal compute, its measured CPU latency is ∼2.68 ms. By comparison, LEEDNet (Raw) has a similar parameter scale (0.253M) and a higher nominal compute (37 MFLOPs); yet, it achieves ∼0.86 ms on the same CPU (Table 4). In our deployment setting, this shows that FLOPs alone do not predict wall-clock latency. LEEDNet’s core relies on depthwise/separable 1D convolutions with contiguous memory access and short, easily fused operation chains, which keep launch overhead and memory traffic low; the slim EEGConformer, even at 7.51 MFLOPs, uses attention-style components (projections, Softmax, layer norms, residuals) that create additional intermediate tensors, resulting in more kernel launches and higher memory movement, which leads to the observed ∼2.7 ms latency.

Overall, the comparisons in Table 4 indicate that LEEDNet provides a good balance between diagnostic performance and efficiency: sub-millisecond GPU and <1 ms CPU inference for the raw branch, and only a few milliseconds for the multi-branch variants. This makes LEEDNet suitable for on-device triage and continuous monitoring in resource-constrained clinical environments.

**Table 4:** Computational efficiency comparison of LEEDNet variants with baseline CNN architectures

			Latency (ms)	
Model	Params (M)	FLOPs (MFLOPs)	CPU	GPU
GoogLeNet	8.48	3010	60.0	36.4
VGG16	138.36	30,960	250.0	43.0
InceptionTime	0.44	350.26	3.45	1.96
EEGConformer	0.34	**7**.**51**	2.68	3.51
LEEDNet (FFT)	0.258	41	1.29	1.26
LEEDNet (Wavelet)	0.257	41	2.04	2.01
LEEDNet (Raw + FFT)	0.511	78	1.65	1.32
LEEDNet (Raw + Wavelet)	0.510	78	2.40	2.07
LEEDNet (FFT + Wavelet)	0.515	82	2.83	2.47
LEEDNet (Raw + FFT + Wavelet)	0.768	120	3.19	2.53
LEEDNet (Raw)	**0**.**253**	37	**0**.**86**	**0**.**85**

Params = trainable parameters; FLOPs = floating-point operations per 2-s segment; Latency = average inference time (batch size 1) on Intel i9 CPU and RTX 3090 GPU

**Fig. 4:**
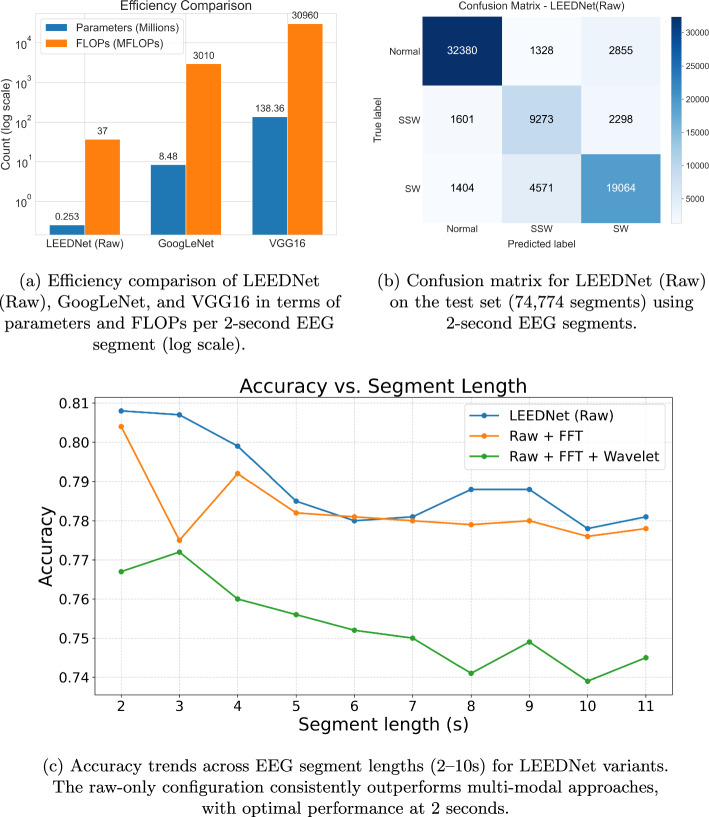
Model efficiency and performance results for LEEDNet

To further assess classification performance, we generated confusion matrices for all evaluated models. Here, we present the matrix for the only best performing configuration—LEEDNet (Raw)—in Fig. 4b. This model shows a strong ability to differentiate among all three classes, with notably low false-negative rates for the clinically critical SSW class.

### Impact of segment length

Segment length plays a critical role in EEG classification, as it determines the temporal context available to the model for detecting clinically relevant patterns. To systematically analyze this effect, we performed an ablation study using segment durations ranging from 2 to 10 s for the top-performing LEEDNet configurations. The results are visualized in Fig. 4c.

Across all evaluated models, performance consistently peaked at 2-second segments. The single-branch LEEDNet (Raw) achieved its highest accuracy of 81.2% at this window size, maintaining stability for approximately 4 s before gradually declining. In contrast, multi-modal configurations such as Raw+FFT and Raw+FFT+Wavelet showed steeper performance drops as the segment length increased.

This trend can be explained by several factors:

1. *Label dilution:* Many pathological EEG events, particularly SW and SSW, are transient in nature and occupy only a small fraction of the recording. As segment length increases, these events make up a smaller proportion of the total samples, causing their discriminative features to be overshadowed by normal background activity.
2. *Increased signal redundancy:* Longer windows introduce repetitive and redundant information, especially in the multi-modal setting. When FFT or wavelet features are fused with raw signals over extended time spans, overlapping information can create noise in the feature space, reducing the model’s ability to focus on truly salient patterns.

Interestingly, the accuracy degradation was more pronounced for multi-modal LEEDNet variants. For example, Raw+FFT dropped from 80.4% (2 s) to 77.5% (10 s), and Raw+FFT+Wavelet fell from 76.6% to 73.3% over the same range. This suggests that while fusion can be beneficial in short windows, extended contexts may exacerbate feature redundancy, leading to diminished generalization.

Overall, our findings reinforce the value of short, localized temporal windows in EEG analysis. A 2-second segment length strikes an optimal balance between providing sufficient context for event recognition and avoiding unnecessary noise or dilution effects. This window size is particularly well-suited for detecting transient abnormalities like SW and SSW, which often span less than a second and require high temporal precision for accurate localization.

## Conclusion and future work

This work introduces LEEDNet, a raw-signal CNN for multi-class, segment-level EEG classification. Across single- and multi-branch variants and three input modalities (raw, FFT, wavelet), the raw-only model delivered the most reliable results on the NMT-Events dataset, attaining 81.22% accuracy and 77.49% macro-F1 with strong sensitivity (78.07%) and specificity (90.76%). Despite offering a richer feature pool, multi-modal fusion with FFT and wavelet representations did not improve performance and often reduced generalization—likely due to redundancy and noise under moderate data regimes. Ablations over segment lengths showed that 2-second windows consistently provide the best trade-off between context and label purity. Longer windows diluted brief pathological events (SW, SSW) with a normal background, reducing separability and accuracy. Practically, these findings recommend a simple recipe for clinical pipelines: to process short, single-channel raw segments with a lightweight CNN and an attention-style temporal gating, and to treat spectral transforms as optional rather than default. The resulting system is easier to train, faster at inference, and easier to interpret (via the fusion gate’s temporal weights), which aligns well with real-time triage and resource-constrained deployments.

Our study uses single-channel crops and a single annotated dataset. Future work will extend LEEDNet to multi-channel EEG for improved spatial modeling and pathological event localization, while also validating the model across diverse clinical datasets and acquisition settings. We also plan to investigate self-supervised pretraining strategies to leverage large-scale unlabeled EEG data, as well as explore hybrid lightweight attention mechanisms that retain computational efficiency like LEEDNet while enhancing long-range temporal modeling. Finally, explainability frameworks and event-level visualization tools will be incorporated to strengthen clinical interpretability and deployment trust.

## Data Availability

This study used the publicly available NMT-Events Dataset. The dataset can be accessed at https://dll.seecs.nust.edu.pk/downloads/. No new data were generated in this work; only analyses of the existing dataset were performed.

## Footnotes

1 https://github.com/dll-ncai/LEEDNet_EEG_Event_Detection
